# MicroRNA-889-3p restrains the proliferation and epithelial–mesenchymal transformation of lung cancer cells via down-regulation of Homeodomain-interacting protein kinase 1

**DOI:** 10.1080/21655979.2021.2000283

**Published:** 2021-11-27

**Authors:** Qiang Zhu, Yun Li, Lina Li, Mingxue Guo, Chenxi Zou, Yi Xu, Zhen Yang

**Affiliations:** a Department of Respiratory Medicine, The First Medical Center of Chinese Pla General Hospital, Beijing, China; b Department of Respiratory Medicine, The Eighth Medical Center of Pla General Hospital, Beijing, China

**Keywords:** MicroRNA-889-3p, lung cancer cells, homeodomain-interacting protein kinase 1, epithelial mesenchymal transition

## Abstract

Dysregulated microRNAs (miRNAs) are common in human cancers and are involved in the proliferation, promotion, and metastasis of tumor cells. Therefore, the aim of this study was to evaluate the expression and biological function of miR-889-3p in lung cancer (LC). MiR-889-3p and Homeodomain-interacting protein kinase 1 (HIPK1) expression was detected in human LC tissues and cells. The correlation of miR-889-3p with the clinicopathology of LC patients was observed. After the transfection of miR-889-3p and HIPK1-related plasmids in human LC cell line A549, several studies were employed for detection of cell growth. In addition, the targeting of miR-889-3p with HIPK1 was verified. The results clarified miR-889-3p was down-regulated, while HIPK1 was up-regulated in LC tissues. Elevated miR-889-3p or repressed HIPK1 weakened the viability, epithelial–mesenchymal transition (EMT), invasion, migration of LC cells, whereas strengthened apoptosis. MiR-889-3p targeted HIPK1; MiR-889-3p mediated HIPK1 to affect the proliferation and EMT of LC cells. Therefore, it is concluded that miR-889-3p repressing HIPK1 restrains the proliferation and EMT of LC cells, providing a novel target for LC therapy.

## Introduction

Lung cancer (LC), the malignant tumor, has the highest mortality rate, with less than 20% survival rate [1]. Its rapid incidence and mortality are key elements in cancer deaths worldwide, causing them one of the most urgent problems to deal with clinically. LC arising from respiratory epithelial cells is assigned into two categories: small cell LC (SCLC) and non-small cell LC (NSCLC). SCLC holding 15% of LC cases is an extremely malignant tumor produced by cells exhibiting neuroendocrine characteristics. NSCLC accounting for 85% is classified into three main pathological subtypes: adenocarcinoma, squamous cell carcinoma and large cell carcinoma [2]. Due to the surprising mortality and morbidity of LC, it is imperative to understand the underlying molecular mechanism of LC, and to develop brand-new prognostic markers and effective therapeutic strategies.

MiRNAs are small and evolutionarily conserved RNAs controlling the mRNA translation process [3]. Plenty of studies have manifested the association of miRNAs with cell metastasis, proliferation, invasion, and apoptosis [4,5]. Due to various epigenetic and genomic changes, miRNAs expression in cancer is obviously unusual [6]. A great many miRNAs serving as tumor suppressor genes or oncogenes block tumor progression, which makes them hopeful targets for cancer therapy [7,8]. Studies have exposed that degradation of miR-889-3p strengthens the invasion, proliferation, and migration of cervical cancer cells and restrains apoptosis [9]. Nevertheless, few studies have been implemented on miR-889-3p in LC.

Homeodomain-interacting protein kinase 1 (HIPK1) is a homologous domain interacting protein kinase, and HIPKs are beneficial for controlling multiple biological processes, such as signal transduction, embryonic development, apoptosis, DNA damage response, and cell proliferation, in response to all kinds of extracellular stimuli [10]. It has been reported that HIPK1 stimulates the epithelial–mesenchymal transition (EMT) in breast cancer cells via activation of the Wnt/β-catenin pathway [11]. Unfortunately, the mechanism of HIPK1 in LC and its EMT have not been fully uncovered.

EMT is a key transition stage from epithelial cells to mesenchymal cells [12]. Tumor invasion and metastasis could be facilitated via changes in EMT phenotype and genes. During EMT, mesenchymal cell markers, such as fibronectin, N-cadherin, and vimentin, are strengthened via cells, while epithelial markers, such as γ-catenin, E-cadherin, and α-catenin, are weakened [13,14].

This study aimed to explore the roles of miR-889-3p and HIPK1 in LC cells and the regulatory mechanism of miR-889-3p/HIPK1 axis in LC cells, thus proposing the hypothesis that miR-889-3p refrained LC cell proliferation and EMT via down-regulating HIPK1.

## 1. Materials and methods

### 1.1 Clinical tissue collection

From 2017 to 2019 within operative period, 61 pairs of human LC matched samples and their matched adjacent non-cancer tissues were gained from the first medical center of Chinese PLA General hospital’s pathology department tumor bank. Matching normal tissue was obtained from 5 cm of the tumor margin and further confirmed by the pathologist. Immediately after resection, the human surgical specimen was frozen in liquid nitrogen and kept in a refrigerator at −80°C. No reception of any treatment in the patients included in this study was conducted prior to resection. The review, improvement and supervision of this study were via the Ethics Committee of the first medical center of Chinese PLA General Hospital (No. z2062). All patients signed written informed consent.

### 1.2 Cells and reagents

The culture of human LC cell lines A549, H1975, H1299, H460, SPC-A1, and normal human lung epithelial cells BEAS-2B [American type culture collection (ATCC), Manassas, VA] was in Roswell Park Memorial Institute (RPMI) 1640 medium containing 10% fetal bovine serum (FBS). RPMI 1640 medium, FBS, and penicillin streptomycin were supplied by Shanghai Jingke Chemical Technology Co., Ltd, and Llipofectamine 2000 via Shanghai Kemin Biotechnology Co., Ltd., Hematoxylin eosin (He) staining kit, and annexin V-fluoresceinisothiocyanat (FITC) kit via BD company, epidermal growth factor receptor (EGFR), and Integrin β1 (ITGB1) antibodies via Abcam.

### 1.3 Cell culture and transfection

Two specific small interfering RNA (siRNAs) for HIPK1 (si-HIPK1#1 and si-HiPK1#2) and one disturbed control siRNA, miRNA mimic (miR-889-3p) and miR-889-3p inhibitor (in-miR-889-3p) as well as negative control miR-NC and in-miR-NC were gained from RIBOBIO (Guangzhou, China). The seeding of cells was conducted into 6-well plates and transfection of the associated plasmids via the Lipofectamine®2000 DNA transfection reagent (Invitrogen, Oregon, USA) [15].

### 1.4 Cell proliferation experiment

The cells in the exponential phase and their suspension were treated with 10 μL Cell counting kit (CCK)-8 solution for 4 h. On the micro-board reader, the measurement of optical density (OD) value was below 450 nm wavelength. The cell proliferation was counted with a blank pore as reference [16].

### 1.5 Plate cloning

In order to carry out colony formation experiment, the seeding of A549 cells (the density of 200 cells per well) was into 12 well plates with culture in Dulbecco’s improved Eagle medium (10% FBS) for 10 d. Colonies were fixed and stained for imaging and quantification [17].

### 1.6 Transwell experiment

The cells (1 × 10^5^) were coated on the top side of polycarbonate transwell filter without Matrigel coating. In the Transwell migration tests, the cells were suspended in a culture medium used with serum in the bottom chamber. After 24-h culture, formaldehyde was fixed for 10 min, and crystal violet staining was employed for 15 min. Cellular numbers were observed under high magnification. The invasion test was the same as the migration test except that the chamber was precoated with Matrigel.

## Apoptosis analysis

The cell Apoptosis Assay Kit (Solarbio, Beijing, China) was applied to evaluate the apoptosis of LC cells. Transfected A549 cells (1 × 10^5^ cells) were stained with Annexin V-FITC and propidium iodide (PI) in 100 μL binding buffer for 15 min and added with 300 μL binding buffer. The cell apoptosis was immediately detected by Attune NxT flow cytometry (ThermoFisher, Waltham, MA, USA) [18].

### 1.7 Real-time quantitative PCR (qPCR)

The extraction of total RNA was conducted from cells with TRIzol reagent (Invitrogen, Carlsbad, CA) obeying manufacturer’s manual. Maxima First Strand cDNA Synthesis Kit (K1642; Thermo Scientific, Shanghai, China) and Maxima SYBR Green/Fluorescein qPCR Master Mix (K0241; Thermo Scientific) were employed for synthesis of cDNA and amplification of special RNA. MiR-889-3p, HIPK1, glyceraldehyde 3-phosphate dehydrogenase (GAPDH) and U6’s qPCR primers are exposed in Table 1. GAPDH and U6 were employed as internal reference to detect RNA levels. The analysis of qPCR data was conducted via 2^−ΔΔCT^ [19].

**Table 1.** Primer sequences

Genes	Primer sequences (5ʹ-3ʹ)
MiR-889-3p	ACACTCCAGCTGGGTTAATATCGGACAAC
	TGGTGTCGTGGAGTCG
U6	GTGCTCGCTTCGGCAGCACAT
	TACCTTGCGAAGTGCTTAAAC
HIPK1 [15]	AGCAGCACCAGTCATCTGTG
	GGAGCTGATGGCCTGAGA
E-cadherin	CGTGAGCATCCAGGCAGTGGTAGC
	GAGCCGCCGCCGCAGGAAG
N-cadherin	CCACCTTAAAATCTGCAGGC
	GTGCATGAAGGACAGCCTCT
GAPDH	AGCCACATCGCTCAGACAC
	GCCCAATACGACCAAATCC

### 1.8 Western blot

After denaturation, the electroblot of 30 μL total protein was placed onto the Polyvinylidene Fluoride (PVDF) membrane by 10% sulfate polyacrylamide gel electrophoresis. After block with fat-free milk, the incubation of these membranes was with the primary antibodies aiming for Phosphorylated AKT (pAkt) (66,444-1-Ig, 1: 1000, Proteintech), Perk (3192, 1: 1000, Cell Signaling Technology), N-cadherin (ab18203, 1: 1000), Vimentin (ab92547, 1: 1000), HIPK1 (ab90103, 1: 1000) and GAPDH (ab8245, 1: 1000) (Abcam) and the second antibody. Then, the development and photographing by electrogenerated chemiluminescence method were manifested. One software was employed for the analysis of the band strength and detection of the relative protein expression [20].

### 1.9 The luciferase reporting experiment

The putative binding sites of miR-889-3p with HIPK1 were exposed via starBase. After the construction, the co-transfection of a wild-type (WT) luciferase reporter vector [HIPK1 3ʹ untranslated region (UTR) WT] and a mutant (MUT) one (HIPK1 3ʹUTR MUT) was implemented into A549 cells with miR-889-3p or miR-NC. Then, dual-luciferase reporter assay system (Promega, Madison, WI, USA) was employed for detection of luciferase activity [21].

## RNA immunoprecipitation (RIP) assay

RIP assay was conducted in A549 cells using an RNA-binding protein immunoprecipitation kit (Millipore, Billerica, MA, USA). In short, RIP lysates were prepared from A549 cells transfected with miR-889-3p mimics or mimics NC, and then immunoprecipitation was performed using 5 μL normal mouse Immunoglobulin G (IgG) or 5 μL Anti-Ago2 antibody, followed by Mana RIP RNA binding protein immunoprecipitation kit. The mRNA levels of miR-889-3p and HIPK1 enriched in the beads were determined by reverse transcription quantitative polymerase chain reaction (RT-qPCR) [22].

## Tumor xenograft experiment

The animal experiment was approved by the Animal Protection and Use Committee of our hospital. Female nude mice aged 4–6 weeks were purchased from Shanghai Experimental Animal Center of Chinese Academy of Sciences and randomly divided into two groups for further study. The stable cell lines were established using miR-889-3p mimic group and NC as well as shRNA or control shRNA targeting HIPK1. The cells or control cells were subcutaneously injected into the flank of nude mice. The tumor volume was calculated as (length × width)^2^ (mm^2^) × 0.5. After 28 d, the tumor was collected and weighed [23].

## Terminal deoxynucleotidyl transferase-mediated dUTP-biotin nick end labeling (TUNEL) analysis

The xenograft tumor sections were dewaxed, hydrated, and incubated with protease K at 37°C for 20 min. Then, the sections were added with 3% H_2_O_2_ and 0.1% TritonX-100 to incubate for 10 min. TUNEL reaction solution was incubated at 37°C for 60 min. After phosphate buffer saline wash, diaminobenzidine and hematoxylin were stained at room temperature for 5 min. Ethanol was applied for dehydration, and xylene was responsible for transparency. The sections were blocked with neutral resin. TUNEL positive cells were observed under a microscope (Olympus, Tokyo, Japan) [24].

## 2.0 Statistical analysis

Statistical software SPSS 21.0 (SPSS, Inc., Chicago, IL, USA) was applied to analyze the data. Kolmogorov–Smirnov test clarified that the data were normally distributed and the results were manifested as mean ± standard deviation (SD). Chi-square test was employed to detect the correlation between miR-899-3p and clinicopathological features of LC patients, with t test for comparison between the two groups, one-way analysis of variance (ANOVA) for comparison among multiple groups, and Fisher’s least significant difference t test (LSD-t) for pair comparison after ANOVA analysis. *P* was a two-sided test, *P* < 0.05 was considered statistically significant.

## Results

The study was conducted to explore the role of miR-889-3p and HIPK1 in LC cells, and the regulatory mechanism of miR-889-3p/HIPK1 axis in LC cells. A series of *in vitro* experiments were conducted and found that miR-889-3p repressed LC cell proliferation and EMT by down-regulating HIPK1. Therefore, in the data, it was the first time to investigate the function and mechanism of miR-889-3p and HIPK1 in LC, providing new insights into the pathogenesis of LC.

## 1. MiR-889-3p is reduced in LC tissues and cell lines

**Figure 1.**
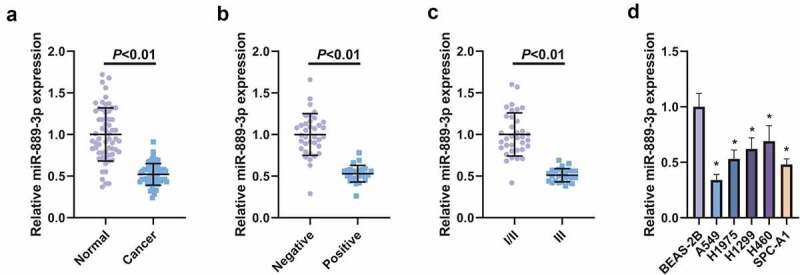
Knockdown of miR-889-3p is manifested in LC tissues and cell lines

With the purpose of detection of the relative expression, miR-889-3p in LC tissues and cell lines was examined in 61 LC and adjacent normal tissues, exposing that miR-889-3p in LC tissue was clearly reduced (Figure 1a). In the meantime, the association of miR-889-3p with clinicopathological features was explored (Table 2), affirming that depression of miR-889-3p was implicated with lymph node metastasis (LNM) and clinicopathological stage (Figure 1b, c). The detection of the relative expression of miR-889-3p was implemented in A549, H1975 H1299, H460, SPC-A1 cells and normal lung epithelial cell BEAS-2B, assuring the decline in LC cell lines versus BEAS-2B cells, and among the cells, the most apparent down-regulation level of miR-889-3p was presented in A549 cells, so A549 cells was selected for the follow-up experiment (Figure 1d). Briefly, repression of miR-889-3p was affirmed in LC tissues and cells.

**Table 2.** Depression of miR-889-3p is associated with LNM and clinicopathological stage

Characteristics	Patients (n = 61)	MiR-889-3p expression		*P*	*χ2*
		Low (n = 30)	High (n = 31)		
Gender				0.300	1.428
Male	36	20	16		
Female	25	10	15		
Age(year)				0.126	2.755
60 or less	33	13	20		
>60	28	17	11		
Differentiation				0.114	2.877
Moderate and good	39	16	23		
Poor	22	14	8		
LNM				0.020*	6.003
Negative	36	13	23		
Positive	25	17	8		
Tumor size				0.300	1.326
<3 cm	37	16	21		
>3 cm	24	14	10		
TNM stage				0.010*	7.096
I/II	35	22	13		
III	26	7	18		

*Indicated statistical significance (*P* < 0.05)

A. MiR-889-3p in LC and adjacent normal tissues analyzed by RT-qPCR; B. Representative association of miR-889-3p with negative or positive LNM; C. Representative correlation of miR-889-3p with tumor stage I/II or III; D. RT-qPCR employed for detection of miR-889-3p in LC cell lines (A549, H1975, H1299, H460, SPC-A1) and BEAS-2B. The data in the figures were all measurement data in the form of mean ± SD. * vs the BEAS-2B, *P* < 0.05.

## 2. MiR-889-3p restrains the proliferation and EMT of LC cells

**Figure 2.**
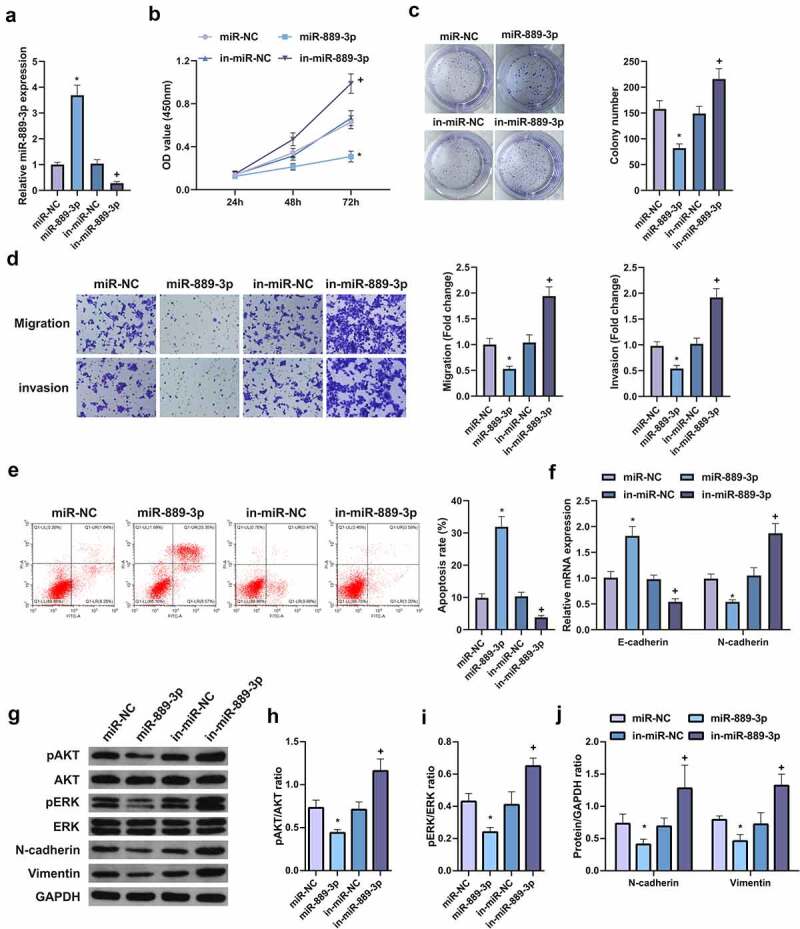
MiR-889-3p represses the proliferation and EMT of LC cells

In order to explore the effect of miR-889-3p expression on the biological behavior of LC cells, A549 cells were selected for the detection of acquired function and lost function. First, the transfection efficiency of the cells was verified by qPCR (Figure 2a), and the cell growth rate was detected by CCK8 method and plate cloning. The results clarified that vs. the control group, the growth rate of A549 cells transfected with overexpressing miR-889-3p was apparently reduced, while miR-889-3p inhibitor elevated the cell growth rate (Figure 2b, c). In addition, cell migration and invasion experiments manifested that miR-889-3p elevation reduced the number of cell migration and invasion 48 h after transfection vs. the control group, while miR-889-3p inhibitor had the opposite effect on A549 cells (Figure 2d, e). Flow cytometry was applied to detect cell apoptosis. It was discovered that up-regulation of miR-889-3p clearly elevated cell apoptosis rate, while inhibition of miR-889-3p reduced cell apoptosis. Subsequently, EMT-related molecules E-cadherin and N-cadherin were detected to evaluate the effect of miR-889-3p on cellular EMT processes (figure 2f). It came out that E-cadherin was elevated but N-cadherin expression was decreased when miR-889-3p was upregulated in A549 cells, while the effect was contrast when miR-889-3p was downregulated. In addition, previous studies have shown that the invasion of HCC cells can be repressed by altering pAKT and pERK signals. The protein levels of pAkt, Perk, NCAD, and Vimentin were detected by Western Blot, finding that overexpression of miR-889-3p clearly reduced pAkt, Perk, N-cadherin, and Vimentin proteins, while repressive miR-889-3p obviously elevated pAkt, Perk, N-cadherin, and Vimentin proteins (Figure 2g). Therefore, these results suggest that miR-889-3p refrains LC cell proliferation and EMT.

A. After transfection of miR-889-3p or in-miR-889-3p, miR-889-3p in A549 cells detected by qPCR; B. The growth rate of cells after transfection of miR-889-3p or in-miR-889-3p detected by CCK-8 method; C. Cell proliferation after transfection with miR-889-3p or in-miR-889-3p examined by plate cloning; D. Migration and invasion of cells after transfection with miR-889-3p or in-miR-889-3p detected by Transwell; E. Flow cytometry employed to detect apoptosis after transfection with miR-889-3p or in-miR-889-3p; F. Detection via qPCR for EMT-related molecules E-cadherin and N-cadherin. G. Western Blot analysis of pAkt, Perk, N-cadherin and Vimentin protein levels. The data in the figures were all measurement data in the form of mean ± SD. * vs the miR-NC, *P* < 0.05; + vs the in-miR-NC, *P* < 0.05.

## 3. MiR-889-3p targets HIPK1

**Figure 3.**
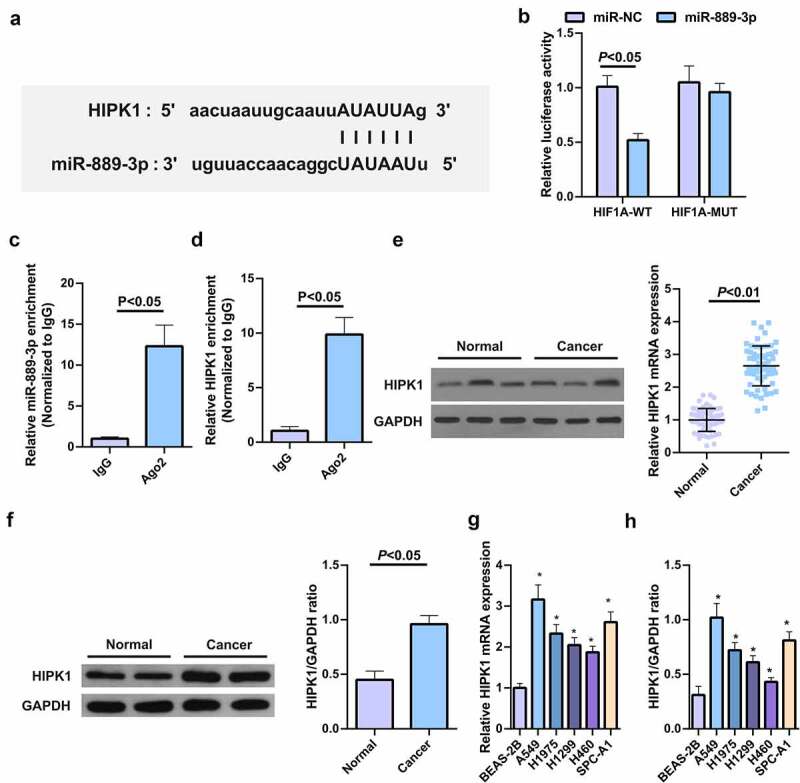
HIPK1 is sponged via miR-889-3p

The binding sites of HIPK1 with miR-889-3p were exposed in StarBase software (Figure 3a), and their mutations resulted in the different luciferase activity of the HIPK1 3ʹUTR MUT reporter vector with the WT vector sensitive to strengthening miR-889-3p. And their luciferase activity was destroyed when transfected with miR-889-3p (Figure 3b). Ago2, as a core component of RNA-induced silencing complex (RISC), is involved in miRNA-mediated mRNA instability or translation inhibition. Therefore, RIP experiment was further conducted via anti-Ago2 antibody, finding that the levels of miR-889-3p and HIPK1 precipitated by anti-Ago2 antibody were clearly elevated vs. IgG group (Figure 3c, d). HIPK1 mRNA was enhanced in human LC tumor tissues and cells (Figure 3e, f). Versus normal tissues and cells, HIPK1 protein in LC cell extracts was up-regulated in general (Fig. 3GH). In brief, elevated HIPK1 is exposed to LC patients and cells and is supposed to be a target of miR-889-3p.

A. The binding sites of HIPK1 with miR-889-3p manifested in StarBase software; B. MiR-889-3p enrichment in A549 cells by Ago2 antibody or IgG; C. HIPK1 enrichment in A549 cells by Ago2 antibody or IgG, and the targeting of miR-889-3p with HIPK1 verified via dual luciferase reporting experiment; D. Detection via qPCR for HIPK1 in clinical samples; E. Detection via qPCR for HIPK1 in cell lines; F. HIPK1 in clinical samples detected by Western Blot; G. HIPK1 in cell lines detected by Western Blot. The data in the figures were all measurement data in the form of mean ± SD. * vs the BEAS-2B, *P* < 0.05.

## 4. HIPK1 depletion depresses LC cell proliferation and EMT

**Figure 4.**
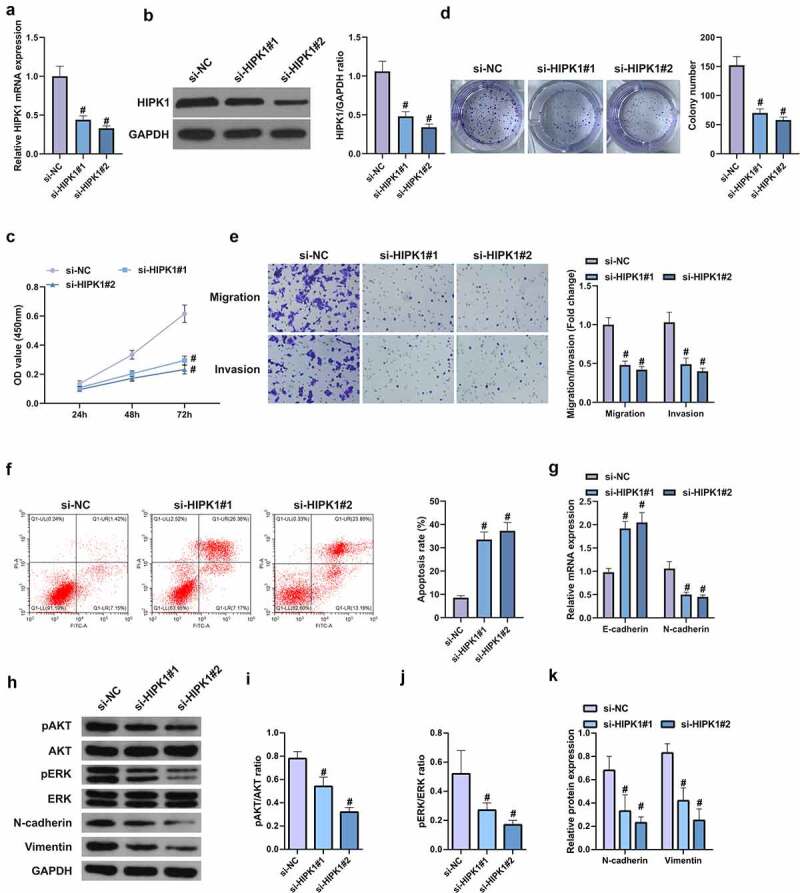
HIPK1 depletion inhibits the proliferation and EMT of LC cells

For further research of the role of HIPK1 on LC, the design of targeted two HIPK1 siRNA was conducted for silencing, which could be seen as a better efficiency in A549 cells caused via si-HIPK1#2 (Figure 4a, b). It was manifested that the OD values of si-HIPK1#1 and si-HiPK1#2 transfected cells were reduced versus si-NC transfected cells within 72 h (Figure 4c). It was then affirmed that the cell migration and invasion rates were continuously repressed after silence of HIPK1, with weakening of proliferation, migration, invasion N-cadherin, pAkt, Perk and Vimentin, and an increase in apoptosis rate and E-cadherin (Figure 4d-h). In a short, HIPK1 depletion curbs the proliferation and EMT of LC cells.

A/B. After HIPK1 silencing, HIPK1 in A549 cells detected by qPCR and Western Blot; C. Cell growth rate after HIPK1 silencing examined by CCK8 method; D. The proliferation of HIPK1 silenced cells detected by plate cloning; E. Cell migration and invasion after HIPK1 silencing detected via Transwell; F. Apoptosis after HIPK1 silencing detected by flow cytometry; G. EMT-related molecules E-cadherin and N-cadherin detected by PCR. H. Western Blot analysis of pAkt, Perk, N-cadherin, and Vimentin protein levels. The data in the figures were all measurement data in the form of mean ± SD. # vs the si-NC, *P* < 0.05.

## 5. HIPK1 overexpression promotes the growth and metastasis of LC cells

**Figure 5.**
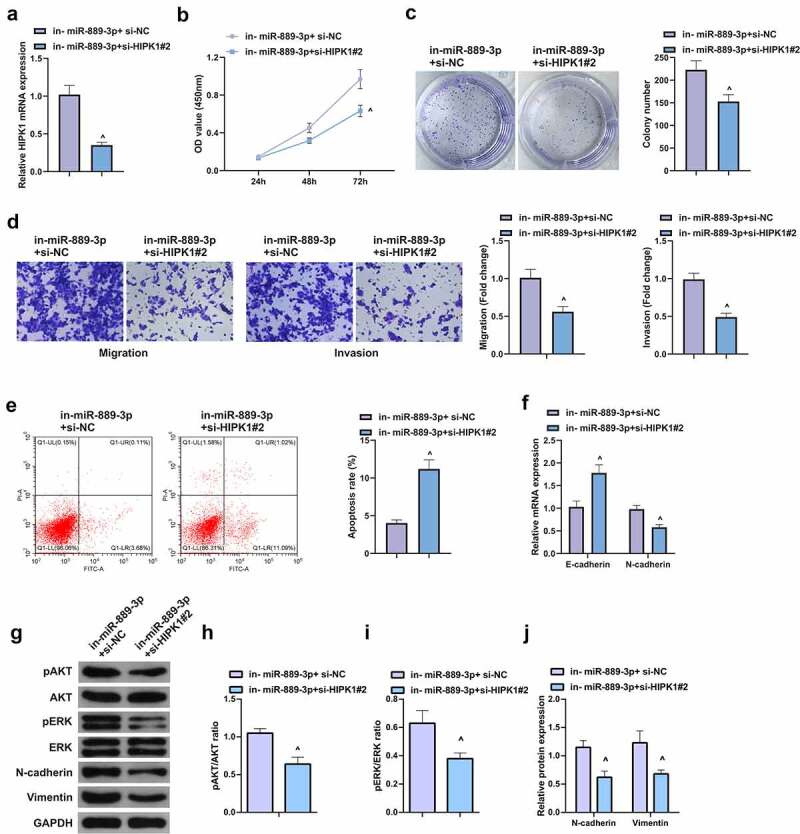
HIPK1 overexpression motivates the growth and metastasis of LC cells

To further verify the effect of HIPK1 on LC cells, HIPK1 was overexpressed in A549 cells (Figure 5a). It was found that in the cells with upregulated HIPK1, the cell proliferation, migration, invasion, and N-cadherin, pAkt, Perk, and Vimentin expression levels were memorably elevated, while apoptosis and E-cadherin expression were clearly decreased (Figure 5b-g), suggesting that HIPK1 overexpression promotes the growth and metastasis of LC cells.

A. qPCR to detect the expression of HIPK1 after elevation of HIPK1; B/C. CCK-8 and plate cloning to detect the proliferation of cells after elevated HIPK1; D. Transwell to detect the invasion and migration abilities of cells after elevated HIPK1; E. Cell apoptosis after elevated HIPK1 detected by flow cytometry; F. EMT-related molecules E-cadherin and N-cadherin detected by PCR; G. Western Blot analysis of pAkt, Perk, N-cadherin and Vimentin protein levels. The data in the figures were all measurement data in the form of mean ± SD. & vs the oe-NC, *P* < 0.05.

## 6. HIPK1 mediating miR-889-3p participates in LC cell proliferation and EMT

**Figure 6.**
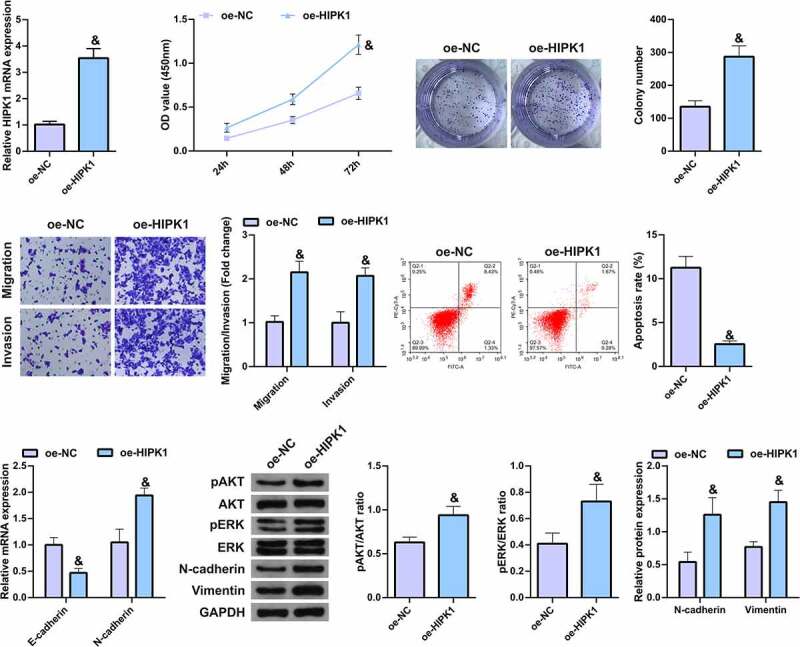
HIPK1-mediating miR-889-3p takes part in LC cell proliferation and EMT

With the purpose of verifying the function of miR-889-3p/HIPK1 axis in LC, a rescue experiment was conducted. Since si-HIPK1#2 showed better silencing efficiency in A549 cells and had a more apparent effect on cells, si-HIPK1#2 was selected for subsequent rescue experiments. A549 cells were co-transfected with in-miR-889-3p and si-HIPK1#2 (Figure 6a), assuring that HIPK1 silence reversed the affected proliferation via miR-889-3p inhibitor (Figure 6b, c). In contrast with the in-miR-889-3p + si-NC, restrained HIPK1 lessened the number of invaded cells, N-cadherin, pAkt, Perk, and Vimentin, but augmented apoptosis rate and E-cadherin (Figure 6d-g). In a short, strengthening HIPK1 is able to reverse the proliferation and EMT of miR-889-3p in A549 cells.

A. Detection via qPCR for HIPK1 in A549 cells; B. The cell growth rate in each group detected by CCK-8 method; C. Cell proliferation in each group detected by plate cloning; D. Cell migration and invasion in each group detected by Transwell; E. Apoptosis in each group examined via Flow cytometry; F. EMT-related molecules E-cadherin and N-cadherin detected via PCR. The data in the figures were all measurement data in the form of mean ± SD. ^ vs the si-NC, *P* < 0.05.

## 7. MiR-889-3p up-regulation or HIPK1 knockdown refrains tumor growth *in vivo*

**Figure 7.**
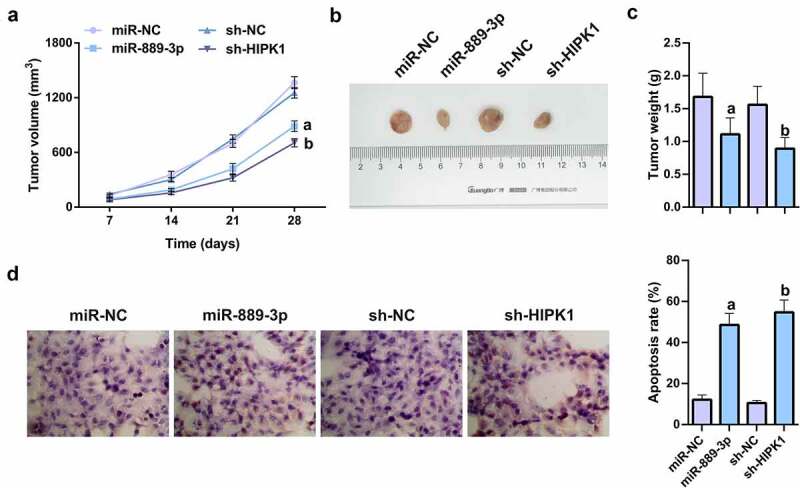
MiR-889-3p upregulation or HIPK1 knockdown represses tumor growth *in vivo.*

To verify the effects of miR-889-3p and HIPK1 on tumor growth *in vivo*, nude mice were injected with the cells transfected with miR-889-3p mimic, HIPK1 shRNA, and their NCs. The results of xenografted tumor in nude mice manifested that miR-889-3p upregulation or HIPK1 knockdown clearly refrained from the volume and weight of subcutaneous tumor (Figure 7a-c). The TUNEL assay on tumor revealed that miR-889-3p enhancement or HIPK1 repression apparently elevated the apoptosis rate of tumor tissue (Figure 7d). In summary, the results suggest that miR-889-3p upregulation or HIPK1 knockdown represses tumor growth *in vivo*.

A. Growth curve images of nude mice after subcutaneous injection of each group of cells; B. Tumor images after 28 days; C. Tumor weight in nude mice 28 days after subcutaneous injection of cells in each group; D. TUNEL staining to detect apoptosis in the tumor tissue of each group. The data in the figures were all measurement data in the form of mean ± SD; a vs the miR-NC, *P* < 0.05; b vs the sh-NC, *P* < 0.05.

## Discussion

Cancer is one of the most severe health problems in the society. LC is the most familiar cancer around the world and the most key cause of cancer-related death. LC, occupying 20% of all cancer-related mortality [25], is the malignant tumor with the highest incidence worldwide. It is featured via a high degree of malignancy, unpleasing prognosis, and surprising mortality. Epidemiological investigation manifests that the occurrence of LC is elevating year by year [26]. Although surgical resection is an effective approach for the treatment of LC, the 5-year survival rate is frustrating owing to the absence of effective early diagnosis means, exposing as rapid drops from about 50% for stage I patients to 20% for advanced patients [27]. No radical resection was conducted in most patients, whose lives only could be prolonged to a certain degree via adjuvant therapies such as radiotherapy and chemotherapy, but the effect is relatively unsatisfying, explained that both imaging techniques and serological markers have relatively poor sensitivity and specificity for the diagnosis of LC [28]. Advanced diagnosis, inefficient chemotherapy agents, unavailability of therapeutic targets, and unreliable biomarkers pose challenges for the treatment of LC [29,30]. Therefore, to improve the prognosis of LC patients, there is an emergency to explore more specific and less invasive diagnostic markers.

MiRNAs, representing a new group of small, noncoding endogenous RNAs, are able to negatively modulate target genes by cutting, disrupting the stability of the target mRNA or prohibiting its translation. New evidence assures that miRNAs are suitable for mediating a series of crucial biological processes and diseases, such as differentiation, apoptosis, cell proliferation, and fibrosis [31]. Like other cancers, tumor suppressor miRNAs in LC also consist of miRNAs whose expression levels are inversely proportional to the rate and severity of cancer progression. Exploring effective approaches to restore the expression of these miRNAs is vital for the treatment and control of cancer or disease complications [32,33]. MiR-889, located at 14q32.31, is associated with pulmonary tuberculosis, esophageal squamous cell carcinoma, and colorectal cancer [34–36]. In the meantime, studies have exposed that miR-889-3p targets FGFR2 to inhibit the activity and invasion of cervical cancer cells [37]. MiR-889-3p accelerates the proliferation of osteosarcoma cells with regulation of cell cycle progression [38]; nevertheless, the character of miR-889-3p in LC has not been fully clarified. In this study, the down-regulation of miR-889-3p was first detected in LC, observing that repression of miR-889-3p in clinic was closely implicated with LNM and clinicopathological stage. Furthermore, the influence of miR-889-3p was investigated on the biological behavior of LC cells. A549 cells were chosen for functional gain and loss detection, assuring that A549 cell growth was obviously restrained via strengthening miR-889-3p, while miR-889-3p inhibitor was a contrast. What is more, during tumor progression, tumor cells will throw out cell–cell adhesion and invade the basement membrane through EMT, and obtain the ability to move, thus forming metastatic secondary tumor lesions [39,40]. Therefore, reversing tumor cell EMT program will be a functional strategy for clinical transformation into cancer therapy later [41]. In this study, through the detection of EMT-related molecules E-cadherin and N-cadherin, the character of miR-889-3p on the cellular EMT process was assessed, affirming that strengthening miR-889-3p accelerated E-cadherin but restrained N-cadherin, which was opposite in knockdown of miR-889-3p. All these evidences confirm that miR-889-3p is available for induction of the apoptosis rate and depletion of the proliferation and EMT of LC cells.

The mRNA is able to block the translation of mRNA to protein and/or degradation of mRNA by combination of the untranslated region of target mRNA [42,43–46], so the online miRNA target prediction software Starbase was applied for prediction of the downstream target genes of miR-889-3p, manifesting that HIPK1 was a potential target of miR-889-3p. Previous studies have suggested that HIPK1 is a carcinogenic gene controlling cell apoptosis and DNA damage repair [4–46], and can stimulate EMT in breast cancer cells via activation of Wnt/β-catenin pathway, but HIPK1 has been little explored in LC. It was found in this study that HIPK1 was up-regulated in tumor tissues of LC patients, and the depletion of HIPK1 had a suppressive effect on the proliferation and EMT of LC cells. Moreover, HIPK1 also mediated miR-889-3p to influence LC cell proliferation and EMT.

## Conclusion

All in all, miR-889-3p interacts with HIPK1 and represses LC. To further understand the molecular mechanism of LC, miR-889-3p/HIPK is expected to become a brand new therapeutic target. The relatively small size of the trial requires further confirmation of the results of a larger study.
